# Production of Protein Concentrates from Macauba (*Acrocomia aculeata* and *Acrocomia totai*) Kernels by Sieve Fractionation

**DOI:** 10.3390/foods11223608

**Published:** 2022-11-12

**Authors:** Sérgio Henrique Toledo e Silva, Lidiane Bataglia Silva, Peter Eisner, Stephanie Bader-Mittermaier

**Affiliations:** 1 Department of Food Process Development, Fraunhofer Institute for Process Engineering and Packaging IVV, 85354 Freising, Germany; 2 TUM School of Life Sciences Weihenstephan, Technical University of Munich (TUM), 85354 Freising, Germany; 3 Steinbeis Hochschule Berlin, 12489 Berlin, Germany

**Keywords:** bocaiuva, oilseed proteins, dry fractionation, plant-based, industrial by-products, functional

## Abstract

Macauba palm fruits (*Acrocomia aculeata* and *Acrocomia totai*) are emerging as sources of high-quality oils from their pulp and kernels. The protein-rich macauba kernel meal (MKM) left after oil extraction remains undervalued, mainly due to the lack of suitable deoiling parameters and integrated protein recovery methods. Therefore, the present study aimed to produce protein concentrates from MKM using sieve fractionation. The deoiling parameters, comprising pressing, milling, and solvent extraction, were improved in terms of MKM functionality. The combination of hydraulic pressing, milling to 1 mm, and the hexane extraction of *A. aculeata* kernels resulted in MKM with the highest protein solubility (77.1%), emulsifying activity index (181 m^2^/g protein), and emulsion stability (149 min). After sieve fractionation (cut size of 62 µm), this meal yielded a protein concentrate with a protein content of 65.6%, representing a 74.1% protein enrichment compared to the initial MKM. This protein concentrate showed a reduced gelling concentration from 8 to 6%, and an increased emulsion stability from 149 to 345 min, in comparison to the MKM before sieving. Therefore, sieve fractionation after improved deoiling allows for the simple, cheap, and environmentally friendly recovery of MKM proteins, highlighting the potential of macauba kernels as a new source of protein.

## 1. Introduction

The quest for new raw materials that can be sustainably exploited is essential for developing bio-based economies and fostering sustainable development. In this context, underexploited tropical palms, such as macauba (*Acrocomia aculeata* and *Acrocomia totai*), present unique potential for the sustainable supply of food and energy [1]. Macauba is an oil-bearing palm member of the Arecaceae family, native to the tropical and subtropical Americas [1]. Macauba palms can grow in marginal soil and are suitable for intercropping and agroforestry systems, while producing 2500 kg of oil per hectare and year on average [1], thus, showing oil productivity to be superior to many oil crops such as canola, peanuts, soybeans, and sunflower [2]. Different from oil palm (*Elaeis guineensis*), which requires a hot humid tropical environment to grow, the macauba palm adapts to different environments, including cooler subtropical and drier semiarid ecosystems [3], and possesses relative tolerance to periods of rain shortages [1]. Therefore, macauba palms can be cultivated in areas where oil palms cannot be produced, resulting in lower risks of a tropical rain forest clearance [4]. Macauba palms also have a high socioeconomic importance due to their wide occurrence [5]. As such, the macauba palm has recently aroused the interest of scientists and agronomists as a new sustainable source of vegetable oil with significant potential for use in the cosmetic, food, and energy industries [1,2,5].

Among the genus *Acrocomia*, the species *A. aculeata* and *A. totai* present the highest economic relevance, owing to their high oil productivity and multipurpose potential to supply oils, proteins, and fibers [6]. *A. aculeata* has a wide geographical distribution, occurring from Mexico to Argentina, and is the most common palm tree in Brazil [7]. *A. totai* only occurs in South America, mainly in Bolivia, Paraguay, central-west Brazil, and Northern Argentina [6]. Differentiation between *A. aculeata* and *A. totai* based on genetic, morphological, and biometric characteristics is discussed elsewhere [3,6,8]. Therefore, understanding the potential of these two species for the production of protein-rich ingredients can provide the framework for their sustainable exploitation.

Both macauba pulp and kernels are the main fractions of industrial interest [9,10,11]. The kernels represent 5–7% of the fruit mass [11,12] and are rich in oil (48.5–55.9% DM), proteins (14.5–30.1% DM), and insoluble fibers (13.1–27.2% DM) [9,10,11,13]. The kernel oil is mainly composed of lauric acid, representing 33–51% of the total fatty acids [11,13,14], thus, highly suitable for cosmetic and food applications. After oil extraction, a protein-rich meal from the macauba kernels is left [15], which is currently used as low-value animal feed or discarded [2]. However, the deoiled macauba kernel meal (MKM) has a high content of essential sulfur-containing amino acids [15] and, thus, could be a novel source of valuable plant proteins.

Conventionally, oil extraction from oilseeds comprises two main steps: pressing and solvent extraction [16,17]. Pressing can be defined as a compression step to press out a liquid from a solid matrix. Two types of presses are commonly used in oil mills: hydraulic and screw presses [16]. The remaining oil in the partially deoiled cake can be extracted with organic solvents [17,18]. Milling can be applied to improve the solvent’s extraction efficiency by disrupting the oil-bearing cells and increasing the specific surface area [16,17]. Treatments, such as cooking, flaking, and expanding, can also be employed to increase the solvent extraction efficiency [17]. Prior research on the processing of macauba kernels focused on mechanical oil extraction through pressing [11] or on deoiling procedures using different solvents, such as hexane [11,19], supercritical CO_2_, or compressed propane [19], partially in combination with ultrasound assistance [20]. Lescano et al. [11] compared the oil extraction yields from macauba kernels after screw pressing and solvent extraction with ethanol, ethyl ether, hexane, acetone, methanol, and petroleum ether employing the conventional Soxhlet method. The oil extraction yields varied from 35.4 to 46.9%. Pressing showed the lowest oil extraction yield, whereas extraction with hexane, petroleum ether, and ethanol showed the highest yields [11]. Trentini et al. [19] evaluated the oil extraction efficiency with compressed propane (8–12 MPa) and supercritical CO_2_ (18–22 MPa). The findings showed that propane and supercritical CO_2_ provided 100% and 93% of oil extraction efficiency, respectively. The defatted macauba kernel meals from propane and CO_2_ extraction did not differ in water- and oil-binding capacities and emulsifying activity compared to the MKM deoiled with hexane [19]. Rosa et al. [20] employed ultrasonic-assisted extraction (UEA) with ethyl ether as the solvent. The oil extraction efficiency in this case was similar to hexane extraction (conventional Soxhlet method), but with a shorter extraction time (45 min compared to 6 h) and 60% less solvent consumption. Despite showing the possibility to extract oil from macauba kernels with different techniques, those studies focused on single oil extraction methods and the evaluation of the oil extraction yields and efficiency. Detailed investigations of the influence of combined techniques, such as pressing and solvent extraction on the properties of MKM proteins, are lacking. Furthermore, as oil extraction can severely impair protein functionality and recovery [21], improved deoiling parameters considering the abovementioned effects are still to be established for converting MKM into functional protein-rich ingredients.

The valorization of MKM proteins also requires the development of suitable protein recovery concepts. Lopes Lessa et al. [22] evaluated the properties of a protein isolate obtained with alkaline aqueous extraction (pH 9.0) and isoelectric precipitation (pH 4.9) from deoiled MKM. The protein isolate showed a 94.9% protein content and 12.6% yield. In terms of functionality, the protein isolate presented least gelling concentration of 14%, and a water- and oil-binding capacity of 0.97 and 1.53 g/g, respectively [22]. Despite showing the possibility to obtain protein isolates from deoiled macauba meals, different protein recovery concepts must be evaluated to leverage the economic production of protein ingredients from macauba kernels. In macauba kernel endosperm, the storage proteins are present in the form of discrete protein bodies [23], similar to those found in seeds from several monocots and dicots [24,25]. The protein bodies can be recovered through dry fractionation techniques, as previously reported for sunflower [26], lupins [27], soybeans [28], and peas [29]. In dry fractionation, milling is applied to detach the protein bodies from starch granules and plant cell wall polysaccharides [30,31,32]. These components are separated by size, density, or charge difference through sieving, air classification, or electrostatic separation, respectively [33]. Such techniques require only small amounts of water and energy, which is associated with a lower environmental footprint than conventional extraction and isolation methods [31,32]. In addition, protein ingredients obtained with dry fractionation can retain native functionality, owing to a lower exposure to heat and chemicals during extraction, isolation, and drying operations [32]. Sieving fractionation is a common method employed to separate particles on the basis of their size [34]. Conventionally, a series of sieves is used with decreasing mesh sizes [31]. Sieve fractionation was employed for the protein enrichment of peas [35], barley [34,36], quinoa [37], chia [38], canola [39], rapeseed [40], and soybean meals [41]. Sieve fractionation is a simple and inexpensive dry processing method, which could potentially be easily integrated into downstream processing after oil extraction [39], thus, presenting a promising prospect for recovering MKM proteins.

Therefore, the present study aimed to obtain protein concentrates through the sieve fractionation of MKM. For this, we adopted a functionality-driven approach to establish the pressing, milling, and solvent extraction conditions, resulting in a high MKM protein functionality. Following this, the influence of such deoiling parameters on the yield and soluble protein content after sieve fractionation trials was determined. In addition, two macauba species, *A. aculeata* and *A. totai*, were used for meal preparation and sieve fractionation to investigate the differences in functionality and fractionation efficiency. After adapting the oil extraction conditions and selecting the most promising macauba species, the composition and functionality of the fractions after a representative sieve fractionation was examined for the selection of cut sizes to produce protein concentrates and the assessment of their application potential as a food ingredient. Therefore, the development of an integrated oil and protein recovery concept based on dry fractionation for macauba kernels is reported for the first time, contributing to the valorization of macauba kernels as a novel source of plant proteins.

## 2. Materials and Methods

### 2.1. Chemicals

Hexane, ethanol, isopropanol, sodium chloride, sodium hydroxide, sodium dodecyl sulfate (SDS), sodium phosphate, sodium dihydrogen phosphate, and copper sulfate were purchased from Synth (Diadema, Brazil). Bovine serum albumin (BSA) and potassium sodium tartrate were purchased from Sigma-Aldrich (Steinheim, Germany). All chemicals were of analytical grade. Refined Liza^®^ soybean oil and Candida^®^ sodium hypochlorite were purchased in a local supermarket.

### 2.2. Macauba Kernels

**Figure 1:**
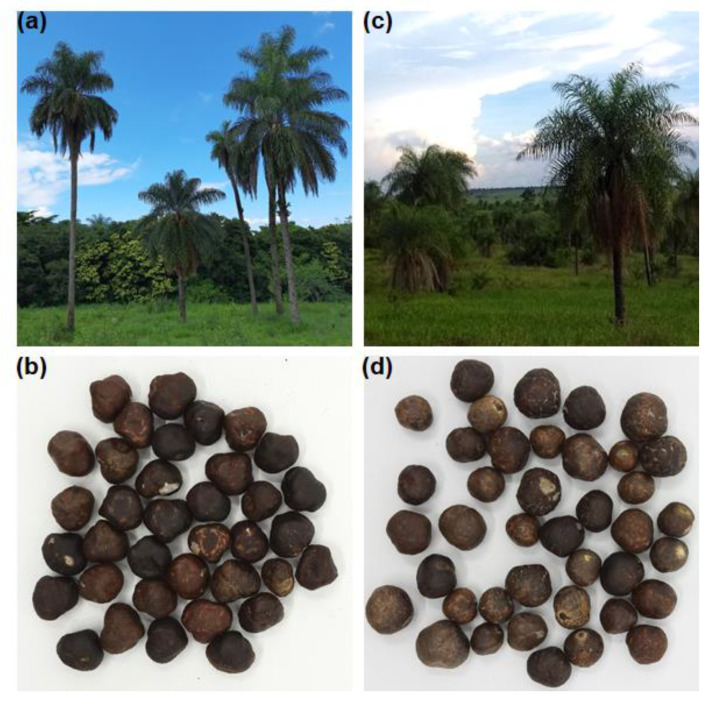
(**a**) Macauba palms and (**b**) kernels of *Acrocomia aculeata*. (**c**) Macauba palms and (**d**) kernels of *Acrocomia totai*.

Macauba fruits were collected from populations of native palms in the state of São Paulo, Brazil. Fruits of *A. aculeata* were harvested in 2017 in the municipality of Dourado. Fruits of *A. totai* were collected during the harvest of 2019 in the city of Presidente Epitácio. Bunches of mature fruits were cut from the mother tree and allowed to fall onto a plastic canvas to prevent contact with the ground. The fruits were selected according to the integrity of the hulls, washed with water, immersed in 200 ppm sodium hypochlorite solution for 5 min, and then washed with water again. The cleaned fruits were dried in a greenhouse under environmental conditions for 2 weeks. After drying, the fruits were mechanically separated into hulls, pulps, and endocarps (inner shell) using a pulper (Saturno, Sete Lagoas, Brazil) operated batch-wise (operation capacity: 20 kg/h). The endocarps were broken using a roller mill (Elipse, Campinas, Brazil), with both rolls operating at 1800 rpm. The integer kernels were manually separated from the broken shells and stored at −20 °C until use. Before processing, the macauba kernels were defrosted overnight at room temperature. The macauba palms and kernels from *A. aculeata* and *A. totai* used in the present studied are depicted in Figure 1. The kernels from *A. aculeata* showed the following composition: 95.1% dry matter (DM), of which 65.5% was oil, 18.5% was the total dietary fibers, 14.3% was protein, and 1.1% was ash. The composition of the kernels from *A. totai* was 95.2% DM, of which 69.2% was oil, 16.3% was the total dietary fibers, 12.1% was protein, and 1.4% was ash. The dry matter and ash contents were determined according to the § 64 LFGB methods at 105 °C and 550 °C, respectively [42]. The oil content was determined with the Soxhlet extraction method using hexane [43]. The total protein content was determined with the Kjeldahl method [44], employing a nitrogen conversion factor of 6.25. The total dietary fiber content was determined following the enzymatic–gravimetric method [45].

### 2.3. Influence of Oil Extraction and Macauba Species on the Quality and Sieve Fractionation of Macauba Kernel Meals

**Figure 2:**
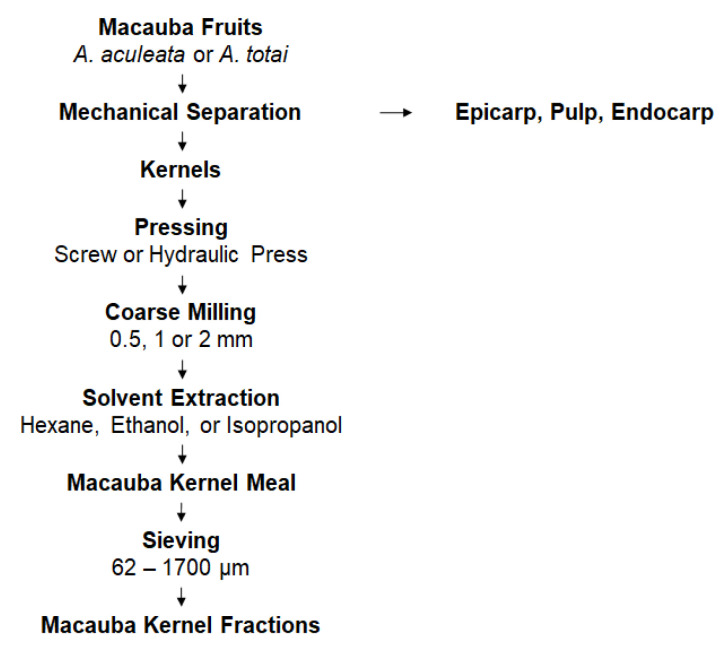
General processing of macauba kernels.

Figure 2 depicts the general processing of macauba kernels. Our approach consisted of a systematic evaluation of the critical oil extraction steps on the characteristics of the protein kernel meals by applying a one-factor-at-a-time model. As such, the influence of the pressing method, milling, and type of solvent on the functionality of MKM proteins and their suitability for sieve fractionation in exploratory sieving trials were determined. In addition, two macauba species were evaluated, *A. aculeata* and *A. totai*, to select the meal with a superior functionality and fractionation efficiency. The tested parameters are listed in Table 1 and described in detail in the following sections.

#### 2.3.1. Oil Extraction and Preparation of Macauba Kernel Meals

In total, 1.5 kg of macauba kernels from *A. aculeata* was pressed for 30 min at 43.5 MPa using a hydraulic press (Mini C, Carver Inc., Wabash, IN, USA) under environmental conditions. Alternatively, 1.5 kg of kernels of *A. aculeata* was screw-pressed using an endless screw extractor ERT 60II (Scott Tech, Vinhedo, Brazil) with a screw diameter of 5.8 cm and a pitch of 25 mm, operating at 48 rpm. The average press cake temperature from screw pressing was 80 °C. The press cakes from both the hydraulic and screw pressing were milled using an impact mill (MF 10.2, Ika-Werke GmbH, Koenigswinter, Germany) with a screen insert of 1 mm. The solvent extraction of the press cakes obtained after hydraulic or screw pressing was accomplished with hexane in a 2.5 L laboratory Soxhlet apparatus. Thereby, 350 g of press cake was used, and the percolation time was set until a residual oil content below 5% (DM basis) was achieved. The MKM was desolventized overnight under a fume hood and labeled as HHA_1_ and SHA_1_ for hydraulic and screw pressing, respectively.

To investigate the impact of milling on protein functionality and sieve fractionation, the press cakes of *A. aculeata* from hydraulic pressing were also milled with screen inserts of 0.5 mm and 2 mm. These ground press cakes were subjected to extraction with hexane as described above and designated as HHA_0.5_ and HHA_2_, respectively. As additional solvents, ethanol and isopropanol were used to extract the hydraulic pressed macauba kernels after milling to 1 mm. The samples were designated as HEA_1_ and HIA_1_ for the ethanol and isopropanol deoiled meals, respectively. To evaluate the influence of macauba species on the protein meal functionality and dry fractionation characteristics, kernels from *A. totai* were hydraulically pressed, milled to 1 mm, solvent-extracted with hexane, and desolventized as described above. The deoiled meal from *A. totai* was designated as HHT_1_.

#### 2.3.2. Sieve Fractionation

The exploratory sieve fractionation of the different types of MKM produced in Section 2.3.1 was performed with the conventional stacked sieve method using a vibrating sieve (model T, Produtest, Bom Retiro, Brazil) operated at 60 Hz and maximum amplitude (position 10 of the device potentiometer) for 30 min. Exploratory sieving was performed in triplicate with 50 g of MKM using seven different sieves with opening diameters of 1700, 1000, 500, 250, 150, 100, and 62 µm and a bottom pan. The sieved fractions were labeled after their corresponding sieves, and the fine fraction collected in the bottom pan was labeled as FF.

A representative sieve fractionation was performed with the MKM from *A. aculeata* obtained after hydraulic pressing, milling to 1 mm, and solvent extraction with hexane. These conditions showed the best results for meal functionality and exploratory sieving performance. The representative sieving was carried out in triplicate with 1 kg of MKM each using a vibrating sieve operated at 60 Hz and maximum amplitude (position 10 of the device potentiometer) for 30 min. The representative sieve fractionation was carried out using the conventional stacked sieve method with seven different sieves (62–1700 µm) and a bottom pan, similarly to the previously described exploratory sieve fractionation. As for the exploratory sieving trials, the sieved fractions were labeled after their corresponding sieves, while the fine fraction collected in the bottom pan was labeled FF. The increase in the MKM amount from 50 g to 1 kg for the representative sieving trial intended to provide sufficient amounts of samples for the analysis of the chemical composition and functional properties.

#### 2.3.3. Determination of Yields and Process Efficiency

The performance of the exploratory sieve fractionation was evaluated through determining the yields of the sieved fractions, which were calculated as described in Equation (1).

(1) $$Y_{i}\left[\%\right]=\frac{F_{i}\left[g\right]}{M\left[g\right]}\cdot 100,$$

where Y_i_ is the yield of the fraction retained in the sieve with opening diameter i, F_i_ is the mass of the fraction retained in the sieve with opening diameter i, and M is the mass of the meal subjected to sieving.

The representative sieve fractionation was assessed through the determination of the process performance parameter yields (Equation (1)), protein separation efficiency (PSE, Equation (2)), and protein enrichment (PE, Equation (3)).

(2) $$PSE_{i}\left[\%\right]=\frac{TPC_{i}\left[g/100g\;DM\right]}{TPC_{M}\left[g/100g\;DM\right]}\cdot Y_{i}\;\left[\%\right],$$

(3) $$PE_{i}\left[\%\right]=\frac{TPC_{i}\left[g/100g\;DM\right]-TPC_{M}\;\left[g/100g\;DM\right]}{TPC_{M}\left[g/100g\;DM\right]}\cdot 100,$$

where PSE_i_ is the protein separation efficiency of fraction i, and TPC_i_ and TPC_M_ are the total protein content in fraction i and the meal, respectively. PE_i_ is the protein enrichment of fraction i.

### 2.4. Analytics

The macauba kernel meal and the sieved fractions were analyzed regarding their chemical composition and functional properties as described below. All analyses were performed in triplicate (*n* = 3).

#### 2.4.1. Composition of the MKM and the Sieved Fractions

The dry matter content was determined at 105 °C until a constant weight was reached [42]. The oil content was determined with the Soxhlet extraction method using hexane [43]. The total protein content was determined with the Kjeldahl method [44], employing a nitrogen conversion factor of 6.25.

#### 2.4.2. Functional Properties of the MKM and Sieved Fractions

##### Soluble Protein Content (SPC) and Protein Solubility (PS)

The soluble protein content represents the protein content in the sample that can be solubilized under specific conditions, whereas the protein solubility represents the ratio of soluble and total protein contents [46,47]. Protein solubility was used to evaluate the solubility of MKM proteins from different oil extraction conditions and the sieved fractions produced after the representative sieving trials. The soluble protein content was employed to compare the functionality and protein content of the sieved fractions from the exploratory sieving trials with the MKM before sieving.

The soluble protein content and protein solubility were determined at 0.5 mol/L NaCl and pH 7.0, which is the condition of maximum solubility of MKM proteins [15], following the method described by Toledo e Silva et al. [15]. In brief, 500 mg of MKM or sieved fractions was dispersed in 25 mL of 0.5 mol/L NaCl solution. The pH was adjusted to 7.0 with a 0.1 mol/L NaOH solution and kept constant for 60 min. Subsequently, the samples were quantitatively transferred into a 50 mL volumetric flask and the volume was completed with a 0.5 mol/L NaCl solution. The content of the volumetric flask was centrifuged at 3300× *g* for 30 min at room temperature. Finally, the supernatant was filtered, and the protein content in the liquid extract was determined with the Biuret method [48] calibrated with BSA.

The soluble protein content and protein solubility were calculated according to Equations (4) and (5), respectively.

(4) $$\;SPC\;\left[g/100g\;DM\right]=\frac{C\left[g/ML\right]\cdot V\left[ML\right]}{M\left[g\right]\cdot DM\left[g/100g\right]}\cdot 100\;$$

(5) $$\;PS\;\left[\%\right]=\frac{SPC\;\left[g/100g\;DM\right]}{TPC\;\left[g/100g\;DM\right]}\cdot 100\;$$

where SPC is the soluble protein content, c is the concentration of protein in the extract, V is the volume of the solution (in our case, 50 mL), m is the mass of MKM or sieved fraction, DM is the dry matter content determined using conventional drying at 105 °C of the MKM or the sieved fraction, PS is the protein solubility, and TPC is the total protein content of the MKM or the sieved fraction determined with the Kjeldahl method [43].

##### Water- and Oil-Binding Capacities (WBC and OBC)

The water-binding capacity (mL water/g DM) was determined according to the American Association of Cereal Chemists standard method 56–30 [49] using deionized water after centrifugation at 1000× *g* at 20 °C for 15 min.

The oil-binding capacity (mL oil/g DM) was determined following the method de-scribed by Muranyi et al. [50] using commercially available soybean oil as the oil phase after centrifugation at 700× *g* and 20 °C for 15 min.

##### Emulsifying Activity Index (EAI) and Emulsion Stability (ES)

The emulsifying activity index and emulsion stability were determined using the turbidimetric method, adapted from Pearce and Kinsella [51]. For this, 2 g of MKM or the sieved fraction was dispersed in 100 mL of sodium phosphate buffer (pH 6.8, 0.1 mol/L) under constant stirring (500 rpm) for 60 min at room temperature. The mixture was centrifuged at 3300× *g* for 30 min and filtered with a black ribbon quantitative paper filter. The soluble protein content in the liquid extract was measured using the Biuret method [48] calibrated with BSA. The supernatant was then diluted with sodium phosphate buffer to a soluble protein content of 1 mg/mL. Emulsions were prepared by adding 10 mL of soybean oil to 20 mL of the sample solution using a high-speed homogenizer (Ultra-Turrax T18 basic, IKA-Werke GmbH, Koenigswinter, Germany) with a homogenization speed of 11,000 rpm for 30 s, followed by homogenization at 20,000 rpm for 1 min. An aliquot of 500 µL of the emulsion was pipetted from the bottom of the emulsion and suspended in 50 mL of 0.1% (*m*/*v*) SDS solution. This was carried out at 0 and 10 min after emulsification. The diluted emulsions were transferred to 2.5 mL polystyrene cuvettes with a pathlength of 1 cm, and the absorbance was measured at 500 nm using a UV/Vis spectrophotometer (Model Lambda 35, PerkinElmer, Llantrisant, UK). The emulsifying activity index and emulsion stability were calculated according to Equations (6) and (7), respectively.

(6) $$EAi\;\left[M^{2}/g\;ProTEin\right]=\frac{\left(4.606\cdot A_{0}\right)}{C\left[g\;\mathrm{protein}/m^{3}\right]\cdot \emptyset \cdot L\left[M\right]}\cdot D,$$

(7) $$ES\;\left[Min\right]=\frac{A_{0}}{A_{0}-A_{10}}\cdot \Delta T\left[Min\right],$$

where EAI is the emulsifying activity index, A_0_ is the absorbance at time 0, c is the concentration of protein before the addition of oil, Ø is the mass fraction of oil in the emulsion, which amounted to 0.33 in our study, l is the pathlength of the cuvette, and d is the dilution factor (100 in our assays). Additionally, ES is the emulsion stability, A_10_ is the absorbance measured at 10 min, and Δt is the time interval between measurements, which was 10 min in our investigations.

##### Least Gelling Concentration (LGC)

The least gelling concentration was determined by adapting the method from Silventoinen et al. [52]. In brief, 0.1 to 1 g of MKM or sieved fraction was suspended in 5 mL of sodium phosphate buffer (pH 6.8, 0.1 mol/L) to yield suspensions with a solid content in the range of 2–20% (*m*/*v*). The suspensions were vortexed for 1 min, heated in a water bath (95 °C/1 h), and cooled down (4 °C/2 h). The least gelling concentration was defined as the concentration at which the sample did not slip after the inversion of the test tube.

### 2.5. Statistical Evaluation of Data

Results were reported as mean values ± standard deviations. In addition, the average values of 2 corresponding groups were compared using Tukey’s test at a significance level of 5% (*p* < 0.05). A statistical analysis of data was performed using OriginPro version 2022b software (OriginLab Corporation, Northampton, MA, USA).

## 3. Results

### 3.1. Influence of Oil Extraction Conditions and Macauba Species on the Functionality of MKM

In our work, oil extraction from macauba kernels comprised of pressing, milling, and solvent extraction, resulting in different types of MKM. These MKMs showed similar proximate compositions in the range of 1.1–5.3% for oil, 50.1–52.4% for the total dietary fiber, 37.2–40.5% for protein, and 3.0–4.2% for ash on a dry matter basis. As the different oil extraction steps could severely affect the MKM proteins, we first adapted the deoiling parameters to prevent a loss of protein functionality. For this, we investigated the influence of pressing (hydraulic and screw pressing), milling (0.5, 1, and 2 mm), and the type of solvent (hexane, ethanol, and isopropanol), on the oil content and protein functionality of MKM. In addition, we also compared MKM from different macauba species (*A. aculeata* and *A. totai*) to assess their suitability for producing protein-rich ingredients. For a systematic assessment, the different oil extraction parameters were varied one at a time and, therefore, were discussed separately. The results are shown in Table 2.

Screw pressing (SHA_1_) resulted in a significantly lower oil content in MKM com-pared to hydraulic pressing (HHA_1_). The higher temperature developed during screw pressing increased oil flowability and reduced the capacity of the solid matrix to absorb oil, thus, contributing to an increased oil expression efficiency [16]. The higher oil expression efficiency of screw pressing compared to hydraulic pressing was also reported for walnuts [53] and pistachios [54]. Hydraulic pressing (HHA_1_) resulted in a MKM with higher protein functionality, evidenced by the higher protein solubility, oil-binding capacity, emulsifying activity index, and emulsion stability compared to the screw-pressed MKM (SHA_1_). The temperature increase during screw pressing, which resulted in a measured press cake temperature of 80 °C in our study, is known to cause protein unfolding and aggregation, resulting in a decreased solubility. Reduced protein solubility was also observed in deoiled meals from rapeseeds [55,56], peanuts [57], and walnuts [53] after screw pressing at temperatures exceeding 70 °C. In addition, aggregated proteins have frequently shown impaired abilities to emulsify and bind oil effectively [58], corroborating our results of a lower emulsifying activity index, emulsion stability, and oil-binding capacity of the screw-pressed MKM (SHA_1_). Therefore, hydraulic pressing was adopted in the present study as a standard pressing condition for evaluating further processing steps, such as milling and solvent extraction.

In the second experimental series, the influence of impact milling on the oil content and the functionality of the MKM was evaluated employing different mill inserts of 0.5 mm (HHA_0.5_), 1 mm (HHA_1_), and 2 mm (HHA_2_), respectively. The mill inserts allowed for the discharge of particles smaller than their opening diameter, resulting in a progressive increase in milling time. After milling, the press cakes were solvent-extracted with hexane with temperatures in the range of 40–60 °C. Eight hours of solvent extraction were employed for the press cakes milled to 0.5 and 1 mm, yielding a comparable oil content in dry matter (*p* > 0.05). The press cake milled to 2 mm had to stay in the Soxhlet extractor for 60 h to obtain a similar oil content in dry matter.

As shown in Table 2, the different milling treatments only significantly affected the emulsion stability and oil-binding capacity of the MKM, but not the protein solubility, water-binding capacity, and emulsifying activity index. As no significant difference in protein solubility was obtained in the MKM from milling to 0.5 mm (HHA_0.5_), 1 mm (HHA_1_), and 2 mm (HHA_2_), we hypothesized that mechanical stress followed by a moderate temperature in solvent extraction resulted in a partial denaturation without protein aggregation. Besides protein solubility, emulsion stability is also influenced by properties such as surface charge, surface hydrophobicity, and the molecular flexibility of protein molecules [46,59]. Such properties are highly influenced by the conformational state of the proteins and, thus, are affected by partial denaturation. The highest emulsion stability was obtained from milling to 1 mm (HHA_1_). The prolonged exposure to mechanical stress during milling to 0.5 mm (HHA_0.5_) might have resulted in an increased protein denaturation, therefore, reducing the emulsion stability of the MKM proteins. A reduced emulsion stability was also reported after the extensive milling of egg phosvitin [60]. Protein denaturation due to extended exposure to heat during solvent extraction might have contributed to the impairment of the emulsion stability and the increased oil-binding capacity of the MKM ground to 2 mm (HHA_2_). Protein denaturation from hot solvent extraction was also reported for walnut proteins [53]. As our goal was to preserve protein functionality for the production of protein concentrates through sieve fractionation, milling to 1 mm was chosen as the milling condition and used for the evaluation of different organic solvents in the third series of experiments.

The oil content of the MKM deoiled with the different solvents was not significantly different (*p* > 0.05), as shown in Table 2. The MKM functionality was influenced by the different solvents (hexane, ethanol, and isopropanol) used for oil extraction in different ways, as shown in Table 2. The water-binding capacity was similar for all tested solvents (*p* > 0.05), whereas oil extraction with ethanol (HEA_1_) and isopropanol (HIA_1_) resulted in a higher oil-binding capacity compared to hexane (HHA_1_). The use of ethanol also significantly reduced the protein solubility of the MKM, whereas similar values (*p* > 0.05) were attained for solvent extraction with hexane (HHA_1_) and isopropanol (HIA_1_). The emulsifying activity index and emulsion stability were higher for extraction with hexane (HHA_1_), followed by extraction with isopropanol (HIA_1_) and ethanol (HEA_1_). Alcohols are protein-denaturing agents, affecting molecular forces such as hydrogen bonding and hydrophobic and electrostatic interactions [61,62]. The reduced protein solubility after extraction with ethanol might be attributed to protein denaturation, resulting in the exposure of the hydrophobic side chains and protein aggregation [21]. A decrease in protein solubility as a result of the denaturing effect of hot ethanol was also reported for corn germ [21,63], soybeans [64], rice bran [65], lupins [66], and rapeseed [40]. The reduced emulsifying activity index and emulsion stability in the MKM extracted with ethanol (HEA_1_) and isopropanol (HIA_1_) could also be attributed to protein denaturation, as was similarly observed for proteins from soybeans deoiled with alcohols [67]. The increased oil-binding capacity of the MKM extracted with ethanol (HEA_1_) and isopropanol (HIA_1_) could be related to protein denaturation and the modification of the meal microcomposition. Ethanol and isopropanol can also coextract polar compounds such as phospholipids, vitamins, simple carbohydrates, polyphenols, lignans, and other bioactive compounds [63,68,69]. In this way, proteins and cell wall polysaccharides can be more available to interact with oil, thus, increasing the oil-binding capacity. Additionally, the lower protein solubility, emulsifying activity index, and emulsion stability of MKM extracted with ethanol (HEA_1_) suggested different degrees of denaturation compared to extraction with isopropanol [70,71]. Moreover, hexane seemed to be superior in terms of the resulting functionality of MKM compared to ethanol and isopropanol. Therefore, hexane was adopted in the present study for the solvent extraction of macauba kernel press cakes.

Following the determination of the best oil extraction conditions, the functionality of MKM was assessed with macauba kernels from *A. totai*. The results were compared to those from *A. aculeata* to evaluate the impact of different macauba species on MKM functionality by employing the improved deoiling conditions. The meals from both macauba species showed similar oil contents (*p* > 0.05), as shown in Table 2. The MKM from *A. aculeata* (HHA_1_) showed a higher protein solubility, emulsifying activity index, and emulsion stability, whereas the meal from *A. totai* (HHT_1_) showed higher water- and oil-binding capacities (Table 2). As described in Section 2.2, the kernels from *A. aculeata* and *A. totai* showed comparable proximate compositions, so the difference in meal functionality might have been related to differences in the composition of the macauba kernel storage proteins arising from genetic variability, which might be addressed in future research.

The difference in the composition of storage proteins was reported for macauba kernels from different regions of Brazil [10,15]. Macauba kernels from *A. aculeata* [15] showed contents of albumins, globulins, prolamins, and glutelins of 9.1, 58.5, 9.8, and 22.5% of the total protein extract, respectively. On the other hand, the content of albumins, globulins, prolamins, and glutelins in macauba kernels from the state of Mato Grosso do Sul (western region of Brazil), the same region in which *A. totai* and its hybrids with *A. aculeata* are found [6], was 5.4, 53.5, 1.1, and 40.0%, respectively [10]. The higher protein solubility, emulsifying activity index, and emulsion stability in the MKM from *A. aculeata* (HHA_1_) could, therefore, be related to its higher content of albumins and globulins, whereas the higher content of glutelins could be responsible for the increased water- and oil-binding capacities in the MKM from *A. totai* (HHT_1_). The difference in functionality due to variability in storage protein composition was also reported for different genotypes of soybeans [72] and peas [73]. Solubility and emulsifying properties are fundamental attributes in the development of new protein ingredients. Protein solubility is strongly related to other functional properties, such as viscosity, gelation, foaming, and emulsification, often being a good index for the application potential of protein ingredients [46,59]. In addition, emulsification is one of the most important processes in the manufacture of processed foods; thus, good emulsifying properties are highly desirable [59]. Taking this into account, the MKM from *A. aculeata* (HHA_1_) showed a higher potential for producing valuable protein ingredients, owing to its superior solubility and emulsifying properties compared to the MKM from *A. totai* (HHT_1_).

### 3.2. Influence of Oil Extraction Conditions and Macauba Species on Exploratory Sieve Fractionation of MKM Proteins

We also investigated the influence of deoiling parameters on the sieve fractionation of macauba kernel proteins. As such, the exploratory sieve fractionation trials aimed at correlating the employed deoiling conditions with the production of fractions with an increased protein content.

For this, the MKMs obtained with different pressing methods (hydraulic and screw pressing), mill inserts (0.5, 1, and 2 mm), types of solvents (hexane, ethanol, and isopropanol), and macauba species (*A. aculeata* and *A. totai*) were subjected to sieving as described in Section 2.3.2. The performance criteria for the exploratory sieving trials relied on the production of fractions with an increased solubility and protein content, as indicated by the soluble protein content. Additionally, the yields and oil content of the sieved fractions were also used to assess the efficiency of the exploratory sieving experiments. The yields demonstrated the influence of the deoiling conditions and macauba species on the relative amounts of the sieved fractions. The oil content showed the influence of the different oil extraction steps on the composition of the sieved fractions and the influence of oil content distribution in the production of fractions enriched in protein. The different MKMs described in Section 2.3.1 and discussed in Section 3.1 were used as the input materials for the exploratory sieving trials.

**Figure 3:**
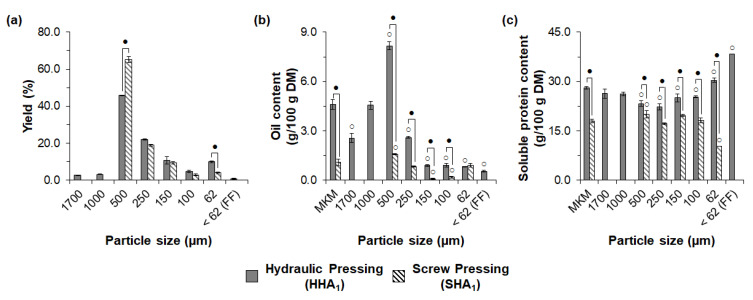
Influence of pressing method on exploratory sieve fractionation of macauba kernel meals after milling to 1 mm and hexane extraction: (**a**) yields, (**b**) oil content, and (**c**) soluble protein content of the sieved fractions. MKM: macauba kernel meal; FF: fine fraction. White circles indicate significant differences (*p* < 0.05) in relation to the respective MKMs. Dark circles indicate significant differences (*p* < 0.05) between fractions of the same particle sizes and different pressing methods.

The pressing method strongly influenced the exploratory sieve fractionation of the MKM. As shown in Figure 3a, eight and five fractions were obtained, respectively, after sieving the MKM prepared through hydraulic (HHA_1_) and screw pressing (SHA_1_), followed by hexane extraction.

The yield of the 500 µm fraction was significantly higher for screw pressing (65.1%) compared to hydraulic pressing (45.8%), whereas the yields of the 100–250 µm fractions were comparable (*p* > 0.05) for the two pressing methods. Moreover, hydraulic pressing (HHA_1_) favored the formation of fractions with smaller particle sizes, evidenced by the significantly higher yield of the 62 µm fraction and the production of the FF (0.8%), which was not obtained after sieving the screw-pressed MKM (SHA_1_). As shown in Figure 3b, the sieved fractions from screw pressing (SHA_1_) showed narrower and significantly lower oil contents (0.1 to 1.6% in DM) compared to the fractions from hydraulic pressing (0.5 to 8.2% in DM), which can be attributed to the higher oil expression efficiency during screw pressing as discussed in Section 3.1.

In terms of solubility, the sieved fractions from hydraulic pressing (HHA_1_) showed a significantly higher soluble protein content than those from screw pressing (SHA_1_), as shown in Figure 3c. The pressing method also influenced the distribution pattern of the soluble protein content after sieve fractionation. For screw pressing (SHA_1_), fractions with a smaller particle size (62–250 µm) showed a significantly lower or similar soluble protein content, whereas the fraction 500 µm showed a significant increase in the soluble protein content compared to meal SHA_1_. For hydraulic pressing (HHA_1_), on the other hand, the fractions with a smaller particle size (62 µm and FF) showed a significantly higher soluble protein content, while the remaining fractions showed a similar or lower soluble protein content compared to meal HHA_1_. The notable increase in the soluble protein content in the FF from hydraulic pressing (from 28.1 to 38.6 g/100 g DM) also suggested an increase in the total protein content, as observed for rice bran proteins dry fractionated using milling and air classification [74]. This highlighted the higher performance of hydraulic pressed MKM (HHA_1_) in exploratory sieving. The inferior sieve fractionation performance from screw pressing (SHA_1_) could be attributed to damage to protein bodies due to high mechanical deformation, as observed for screw-pressed soybean meal subjected to electrostatic separation [28].

Sieving, air classification, or electrostatic separation were employed for the dry fractionation of proteins from pressed meals from rapeseeds [26], canola [39], sunflower seeds [75], and soybeans [28]. Despite showing the feasibility of the dry separation of proteins from different oilseeds, those studies focused on the total protein content of the fractions without assessing the protein functionality or denaturation of the protein-enriched fractions. In addition, a comparison of different pressing methods on the dry fractionation of oilseed meals was not provided, so their influence on the dry recovery of proteins could not be established. Nevertheless, our results clearly showed a strong influence of the pressing method on the sieve fractionation performance of the MKM. Future research on the milling behavior and morphology of protein bodies can contribute to understanding the differences in the dry fractionation behavior of MKMs produced with different pressing methods.

**Figure 4:**
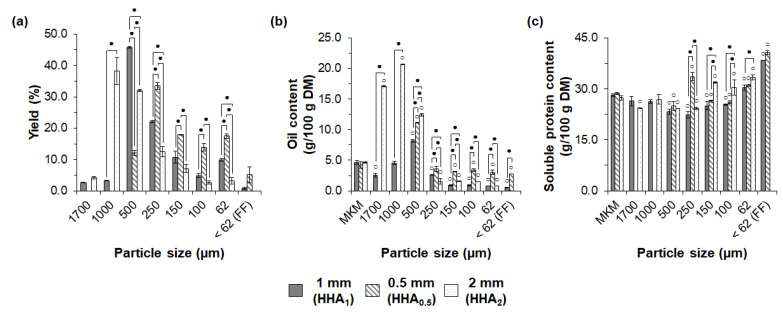
Influence of milling on exploratory sieve fractionation of macauba kernel meals from hydraulic pressing and hexane extraction: (**a**) yields, (**b**) oil content, and (**c**) soluble protein content of the sieved fractions. MKM: macauba kernel meal; FF: fine fraction. White circles indicate significant differences (*p* < 0.05) in relation to respective MKMs. Dark circles indicate significant differences (*p* < 0.05) between fractions of the same particle sizes and different milling conditions.

The sieve fractionation of MKM was also strongly affected by the applied milling condition. As shown in Figure 4a, six, eight, and seven fractions were produced from MKMs after hydraulic pressing, milling to 0.5 mm (HHA_0.5_), 1 mm (HHA_1_), and 2 mm (HHA_2_), respectively, followed by hexane extraction and sieving. As expected, decreasing the aperture of the mill screens from 2 to 0.5 mm shifted the yields of the fractions towards smaller particle sizes. The main fraction after milling to 2 mm (HHA_2_) was 1000 µm (38.2%), whereas the main fractions from milling to 1 mm (HHA_1_) and 0.5 mm (HHA_0.5_) were 500 µm (45.8%) and 250 µm (33.5%), respectively. Milling to 0.5 mm (HHA_0.5_) also significantly increased the yields of the 62–150 µm fractions. The yield of the FF was comparable (*p* > 0.05) after milling to 0.5 mm (HHA_0.5_) and 1 mm (HHA_1_), and was not present using a mill insert of 2 mm (HHA_2_).

As discussed in Section 3.1, the MKMs processed with different mill inserts showed similar oil contents (*p* > 0.05). However, a broader oil content in the sieved fractions was obtained after milling to 2 mm (1.5 to 26.7%), followed by milling to 0.5 mm (2.7–11.1%) and 1 mm (0.5–8.2%), as shown in Figure 4b. In all cases, the coarser fractions (500–1700 µm) showed a significantly higher oil content, which could be attributed to diffusive constraints of the solvent miscella inside larger particles [76].

Additionally, the fractions in the range of <62 µm (FF) to 250 µm from milling to 0.5 mm (HHA_0.5_) showed a significantly higher oil content compared to the same fractions from other milling conditions. In this case, extensive milling might have increased the rupture of oleosomes, resulting in a more homogeneous distribution of oil among the sieved fractions. The lowest oil content was obtained in the FF from milling to 1 mm (HHA_1_).

The distribution of the soluble protein content was also influenced by the applied milling condition, as shown in Figure 4c. For milling to 2 mm (HHA_2_), the 62–150 µm fractions showed a slight, but still significant, increase in the soluble protein content (from 27.3 g/100 g DM in the HHA_2_ to 33.4 g/100 g DM), while the remaining fractions showed a lower or similar soluble protein content compared to the HHA_2_. Milling to 0.5 mm (HHA_0.5_) resulted in 150 µm, 62 µm, and FF fractions with an increased soluble protein content compared to the HHA_0.5_. In the case of milling to 1 mm (HHA_1_), only the 62 µm and FF fractions showed an increased soluble protein content compared to the HHA_1_. The FF from milling to 0.5 mm (HHA_0.5_) and 1 mm (HHA_1_) showed a comparable soluble protein content (*p* > 0.05) in the range of 38.4–40.6 g/100 g DM, which was the highest value among all obtained sieved fractions, representing an almost 1.5 times higher protein content compared to the input materials HHA_0.5_ and HHA_1_.

In dry fractionation, milling promotes the disentanglement of kernel constituents, such as protein bodies, starch granules, and cell wall polysaccharides. After milling, these components can be separated owing to their size, density, or charge difference with separation techniques such as sieving, air classification, or electrostatic separation [31,32]. The influence of the milling extent was reported on for the dry fractionation of peas [29], lupins [27], soybeans [28], and canola [39]. In contrast to our work, these studies focused on the total protein content of the fractions obtained after dry processing. For instance, insufficient milling reduced the yields of the protein-enriched fractions, while extensive milling decreased the purity of the protein-enriched fractions, owing to the presence of fine fiber particles and broken starch granules [29,32]. In these studies, optimum milling was defined as a balance between the yield and purity of the protein-enriched fractions and depended on the raw material being processed. Fine-impact milling (D_0.5_ < 12.9 µm) was employed for the air classification of peas [29], while coarse-impact milling was defined as the optimum condition for the air classification of lupins (D_0.5_ < 100 µm) [27] and the electrostatic separation of soybeans (D_0.5_ < 48.8 µm) [28]. In contrast, Mejicanos et al. [39] observed that hammer milling to 1 or 2 mm jeopardized the sieve fractionation of deoiled canola meals.

In our study, milling to 2 mm (HHA_2_) resulted in the lowest sieve fractionation performance, which might have been attributed to the insufficient disentanglement of the macauba kernel constituents. This was supported by the higher yields of coarse fractions (>500 µm) without the production of a FF. Milling to 1 mm (HHA_1_) resulted in a superior exploratory sieve fractionation performance, evidenced by the formation of the FF with the highest level of enrichment in soluble protein. In this case, the improved sieve fractionation might have been related to a higher level of the disentanglement of the protein bodies and a reduced cohesiveness of the particles from the low oil content in the FF [77]. Further milling to 0.5 mm (HHA_0.5_) did not improve the sieving performance, with a similar yield and soluble protein content of FF compared to milling to 1 mm (HHA_1_). Additionally, the MKM from milling to 1 mm presented the highest MKM functionality (discussed in Section 3.1) compared to the other milling conditions. As our work aimed to obtain functional protein concentrates through sieve fractionation, milling to 1 mm was chosen as the best milling condition.

**Figure 5:**
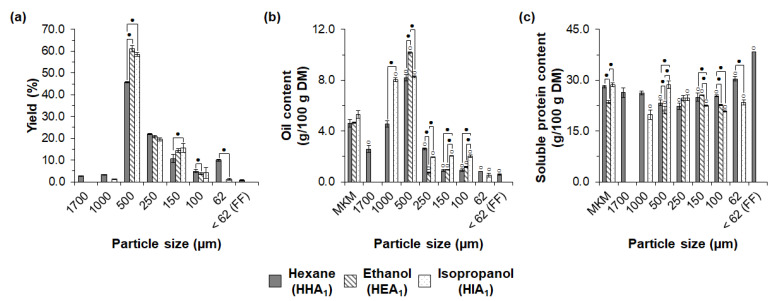
Influence of alternative organic solvents on exploratory sieve fractionation of macauba kernel meals from hydraulic pressing and milling to 1 mm: (**a**) yields, (**b**) oil content, and (**c**) soluble protein content of the sieved fractions. MKM: macauba kernel meal; FF: fine fraction. White circles indicate significant differences (*p* < 0.05) in relation to respective MKMs. Dark circles indicate significant differences (*p* < 0.05) between fractions of the same particle size and different solvent extraction.

The use of ethanol and isopropanol considerably affected the exploratory sieve fractionation of the MKM. As shown in Figure 5a, eight, four, and six fractions were obtained after sieving the MKM deoiled with hexane (HHA_1_), ethanol (HEA_1_), and isopropanol (HIA_1_), respectively. The main fraction for all the tested solvents was 500 µm, with a significantly lower yield for extraction with hexane (45.8%) and similar yields (*p* > 0.05) for deoiling with ethanol (61.2%) and isopropanol (58.4%).

The use of ethanol and isopropanol jeopardized the yield of fractions with small particle sizes. Deoiling with ethanol (HEA_1_) did not produce fractions below 100 µm, and the yield of the 62 µm fraction was significantly lower after extraction with isopropanol (1.1%) compared to hexane (9.0%). In addition, deoiling with hexane (HHA_1_) also produced a FF, which was not obtained after extraction with ethanol (HEA_1_) or isopropanol (HIA_1_).

As shown in Figure 5b, the sieved fractions with large particle sizes showed significantly higher oil content when ethanol and isopropanol were used for oil extraction. The 1000 µm fraction showed oil content of 4.6 and 8.1% after extraction with hexane (HHA_1_) and isopropanol (HIA_1_), respectively. The oil content of the 500 µm fraction was significantly higher for extraction with ethanol (10.3%) than with hexane (8.2%). The lower efficiency of ethanol and isopropanol in deoiling coarser fractions might relate to their lower miscibility with oil [18,78] and diffusive constraints of the solvent miscella in the solid matrix and bulk solution [76].

The soluble protein content of the sieved fractions was also influenced by the type of solvent used for oil extraction (Figure 5c). As previously discussed, the FF from hexane extraction (HHA_1_) showed an increased soluble protein content (38.4 g/100 g DM), indicating a higher functionality and protein enrichment. After deoiling with ethanol (HEA_1_), the 150 µm fraction showed a slight significant increase in the soluble protein content (from 23.5 to 25.6 g/100 g DM) compared to the HEA_1_. When the press cake was extracted with isopropanol (HIA_1_), no fraction showed an increased soluble protein content compared to the input material HIA_1_.

The lower sieve fractionation performance after extraction with ethanol and isopropanol might have been attributed to their denaturing effect, as evidenced by the lower MKM protein functionality, as discussed in Section 3.1. To the best of our knowledge, no studies focusing on the influence of alternative organic solvents in the dry fractionation of oilseed proteins are available. Nevertheless, our results suggested that the solvent employed for deoiling can modify the milling behavior, distribution of nutrients, and functionality of the fractions during dry processing. Therefore, in our study, hexane was the preferred solvent for oil extraction, aiming the dry recovery of macauba kernel proteins. In addition, future research might need to focus on finding out the extraction conditions based on alcohols that largely prevent protein denaturation and improve the dry fractionation of deoiled meals. The determination of meal morphology, the integrity of protein bodies, and protein functionality after solvent deoiling can guide the design of appropriate extraction conditions with alternative organic solvents. This is of strategic importance as alcohols are less toxic and more environmentally friendly than hexane and other petroleum-derived solvents.

**Figure 6:**
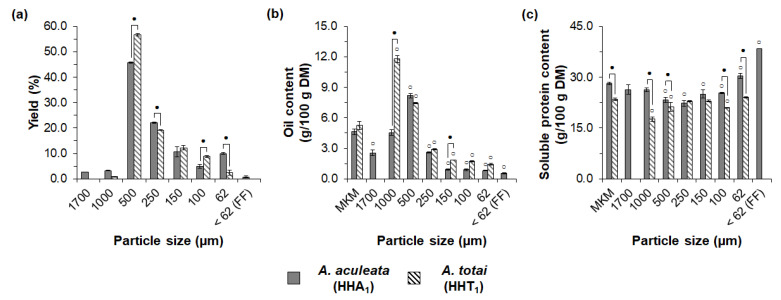
Influence of macauba species on exploratory sieve fractionation of the macauba kernel meals from hydraulic pressing, milling to 1 mm, and solvent extraction with hexane: (**a**) yields, (**b**) oil content, and (**c**) soluble protein content of the sieved fractions. MKM: macauba kernel meal; FF: fine fraction. White circles indicate a significant difference (*p* < 0.05) in relation to respective MKMs. Dark circles indicate a significant difference (*p* < 0.05) between fractions of the same particle sizes and different macauba species.

The meals from the different macauba species also differed in sieve fractionation behavior, as shown in Figure 6. After sieving, the MKM from *A. totai* (HHT_1_) produced six fractions, whereas eight fractions were obtained from *A. aculeata* (HHA_1_). The main fraction from both species was 500 µm, with yields of 45.8% for *A. aculeata* (HHA_1_) and 56.7% for *A. totai* (HHT_1_). Significant differences were also observed in the yields of the 62, 100, and 250 µm fractions. Moreover, *A. aculeata* (HHA_1_) also produced a FF, which was not obtained after the exploratory sieving of *A. totai* (HHT_1_).

For both macauba species, the sieved fractions with the large particle sizes showed a higher oil content, with 11.8% for the 1000 µm fraction from *A. totai* (HHT_1_) and 8.2% for the 500 µm fraction from *A. aculeata* (HHA_1_), as depicted in Figure 6b. As the particle size decreased, the oil content decreased, with the lowest values being 0.55% for the FF from *A. aculeata* (HHA_1_) and 1.44% for the 62 µm fraction from *A. totai* (HHT_1_). As previously discussed, the higher oil content in the coarser fractions could be related to the diffusive constraints of the solvent miscella inside large particles. In addition, the 62, 100, 250, and 500 µm fractions from *A. totai* (HHT_1_) showed a comparable oil content (*p* > 0.05) in relation to the fractions of the same particle sizes from *A. aculeata* (HHA_1_). Therefore, a similar oil content distribution pattern was obtained after the sieve fractionation of MKMs from different macauba species.

Despite similar oil content distributions, the sieved fractions from *A. aculeata* (HHA_1_) and *A. totai* (HHT_1_) differed in the distribution of the soluble protein content (Figure 6c). As previously discussed, the FF from *A. aculeata* (HHA_1_) showed an increased soluble protein content, suggesting an increased total protein content. The fractions from *A. totai* (HHT_1_) showed a similar or lower soluble protein content compared to their MKM (HHT_1_), with no fraction enriched in soluble protein. Therefore, besides a higher meal functionality, the MKM from *A. aculeata* (HHA_1_) also showed a higher potential for protein enrichment using sieve fractionation.

The influence of the genetic variability was reported for the dry fractionation of quinoa [79], wheat [80], barley [80], and deoiled canola meals [39]. Different species, varieties, and cultivars can differ in seed hardness and cellular microstructure [81], potentially modifying milling behavior, and, therefore, the detachment of the seed components during dry processing [30]. Seed hardness can be influenced by the content, composition, and molecular structure of insoluble polysaccharides [81], the adhesion of the protein matrix to other seed structures, the continuity of the protein matrix, and the net charge of the proteins [82]. Therefore, further studies on the composition, microstructure, and tissue architecture of kernels from *A. aculeata* and *A. totai* could assist in understanding the differences in dry fractionation behavior between these species. Nevertheless, our results showed that *A. aculeata* had a higher potential for producing protein-rich products, owing to its higher meal functionality (discussed in Section 3.1) and exploratory sieve fractionation performance. Therefore, the present investigation paves the way for addressing future agricultural advancements of *A. aculeata* in relation to cultivar development, growth conditions, stages of maturity, and postharvest treatments, aiming at the integrated recovery of oil and proteins from macauba fruits.

### 3.3. Representative Sieve Fractionation of Macauba Kernel Proteins

In the previous sections, oil extraction from macauba kernels was adapted to yield MKM with high functionality for the production of protein concentrates through sieve fractionation. Hydraulic pressing, followed by impact milling to 1 mm, and solvent extraction with hexane resulted in the highest MKM functionality and fractionation performance in exploratory sieving trials. These conditions were replicated in a representative sieving trial, employing kernels of *A. aculeata*, owing to its higher potential for producing protein ingredients. The composition (total protein and oil contents) and functionality of the fractions were examined in detail, and the fractionation performance and potential application as a food ingredient were discussed below.

#### 3.3.1. Composition of the Fractions and Sieving Performance after the Representative Sieve Fractionation

The dry fractionation of proteins can be characterized by four parameters: the total protein content, yield, protein separation efficiency, and protein enrichment [31]. The yields represent the mass percentage of original MKM that was recovered into the sieved fractions. The protein separation efficiency is the percentage of the protein from the MKM recovered in the sieved fractions. The protein enrichment denotes the percentage increase in the protein content compared to the input MKM. These parameters were used to assess the performance of the representative sieve fractionation, as shown in Table 3.

Six fractions in the range of <62 µm to 500 µm were obtained after the representative sieving of the MKM prepared with improved oil extraction parameters. The 500 µm fraction showed the highest yield, whereas the yields of the fractions until a particle size of 62 µm were gradually reduced, showing the reproducibility of our exploratory sieving trials. However, the yield of the FF (<62 µm) was greatly increased from 0.8 to 9.4%, and no fractions above 500 µm were obtained, which could be attributed to a better milling performance when the mill was operated with a higher amount of press cakes. Furthermore, the oil content of the MKM and sieved fractions was comparable to the values obtained in the exploratory sieving experiments, showing the reproducibility of the deoiling process.

The total protein content of the MKM was similar to values reported for deoiled macauba kernels [10,15,22]. Compared to the MKM, the fractions between 62 and 500 µm showed a significantly lower total protein content and negative values of protein enrichment, showing that those fractions were depleted in protein. On the other hand, the FF showed the highest protein content, with a notable positive enrichment factor of 74.1%. Protein concentrates are products classified with a protein content of 50–70% [83], therefore, the FF can be called a protein concentrate. Total protein contents between 51 and 59% were reported for protein concentrates from peas [29], lupins [27], beans, and lentils [81] obtained through the use of milling and air classification. The sieve fractionation of rapeseed [40], canola [39], and soybean meals [41] resulted in total protein contents of 58.4, 41.6, and 55.7%, respectively. Therefore, the our process concept proved to be a feasible technique for producing protein concentrates with satisfactory protein contents in a simple, cheap, and environmentally friendly manner. Our results also established a cut size of at least 62 µm for the direct production of protein concentrates from deoiled MKMs using sieve fractionation.

As expected, the highest protein separation efficiency was obtained for the 500 µm fraction, due to it having the highest yield, despite a negative protein enrichment. Liu et al. [34] also reported the highest protein separation efficiency for the fraction with the highest yield after milling and sieving barley seeds. The FF, in turn, showed the third highest value of protein separation efficiency, which could be attributed to its high total protein content and moderate yield compared to the other fractions. However, the protein separation efficiency of the FF was lower compared to the values obtained for the dry fractionation of legume seeds (77–90%) [31] and cereals (26–45%) [84], showing that there was still room for the improvement of the dry separation efficiency of macauba kernel proteins. This could be achieved through the recycling of the protein-depleted fractions with milling and sieving [26,74].

Additionally, the 500 µm fraction could be further deoiled to values below 5%, which is considered adequate for the dry fractionation of plant proteins [81]. Additional techniques, such as air classification or electrostatic separation, can also be applied to improve the recovery efficiency of MKM proteins. These techniques employ different fractionation driving forces, which can improve the selective separation of the protein bodies [32], thus, increasing the total protein content and protein separation efficiency of the FF and, therefore, might need to be addressed in future research.

#### 3.3.2. Functionality of the Sieved Fractions after the Representative Sieve Fractionation

As shown in Table 4, considerable differences in functional properties were observed between the sieved fractions and the MKM. Such differences could be attributed to the difference in the composition of the fractions. Besides the difference in the total protein and oil contents, dry fractionation can also change the distribution of other nutrients, such as the content of starch, dietary fibers, and ash [37,81,85]. The functionality of the obtained fractions was, therefore, strongly related to their exact composition, which should be considered next to the protein and oil contents [32]. As our goal was to obtain protein concentrates through sieve fractionation, the functionality aspects of the FF fraction were discussed in detail to assess its potential as a novel food ingredient.

Gelation is one of the most important functional properties of protein ingredients [46]. The gelling capacity of proteins can impart the desired texture, stability, and flavor perception properties to food products [86]. In our work, the gelling properties of the macauba kernel proteins were probed through the least gelling concentration, in which reduced values represented an increased gelling capacity. As can be seen from Table 4, the FF showed an increased gelling capacity compared to the MKM, which could be attributed to its higher protein content. In addition, the high level of protein enrichment in the FF could also explain its low water- and oil-binding capacities, as a reduced dietary fiber content in this fraction was probably realized, while the denaturation of proteins was decreased by applying a mild treatment during the deoiling.

The emulsifying properties are another key functionality of protein ingredients [83]. The emulsifying properties of the macauba kernel proteins were assessed by measuring the emulsifying activity index and emulsion stability. Compared to the MKM, the FF showed a higher emulsion stability, but a lower emulsifying activity index. Therefore, the FF exhibited an improved capacity to prevent the flocculation and coalescence of the emulsified oil droplets, but with a lower ability to reduce the interfacial tension of the oil–water interface [51]. In the present study, both the emulsifying activity index and emulsion stability were determined with the same soluble protein concentration in the emulsion system’s continuous phase. Therefore, the observed difference in emulsifying properties was more likely related to a difference in the protein composition rather than a difference in the total protein content of the fractions. The protein solubility values of the sieved fractions also suggested that their protein composition was affected by the sieve fractionation. The highest protein solubility was obtained in the 500 µm fraction, which gradually decreased, with the lowest value for the FF. Different compositions of protein subunits were reported for lupin protein concentrates obtained with milling and air classification [27]. The protein concentrate (protein-enriched fraction) showed a higher content of subunits of β-conglutin, while the protein-depleted fraction was richer in albumin subunits [27]. Therefore, determining the exact protein composition of the FF could contribute to understanding the differences in functional properties in relation to the MKM, which might need to be addressed in the future. Nonetheless, our results indicated that the sieve fractionation of macauba kernel meal resulted in a protein concentrate with an improved functionality regarding gelling and emulsion stability properties.

The improvement of the functional properties of protein concentrates obtained using milling and air classification was also reported for peas [29], lupins [27], rice bran [74], and barley [52]. The same was reported for protein-enriched fractions using sieve fractionation for rapeseed [40] and quinoa [37]. Additionally, the FF showed functional properties within the range of protein concentrates obtained through dry fractionation from legume seeds [87,88], cereals [52,74], pseudocereals [37,38], and oilseeds [40]. The FF also showed higher water- and oil-binding capacities and lower least gelling concentrations compared to the macauba kernel protein isolate produced through the use of conventional extraction and isoelectric precipitation [22]. This highlighted the potential of the macauba kernel protein concentrate as a new food ingredient, especially in applications requiring emulsion stabilization and gelling properties, such as in pickering emulsions, meat alternatives, dressings, and dairy desserts [83]. Therefore, sieve fractionation following improved oil extraction is a feasible technique for producing protein concentrates from macauba kernels for a wide range of potential applications.

## 4. Conclusions

Macauba kernels are a potential new source of vegetable oil and plant proteins. The influence of the deoiling steps comprising pressing, milling, and solvent extraction on the functional properties of MKM was systematically investigated. Kernels from two macauba species, *A. aculeata* and *A. totai*, were also screened regarding their potential for producing functional protein ingredients. All oil extraction steps affected the functionality of the macauba kernel proteins. A temperature increase during screw pressing resulted in protein denaturation and jeopardized the protein solubility, oil-binding, and emulsifying properties. Extensive milling also resulted in partial protein denaturation, reducing the emulsion stabilization properties of the macauba kernel meal. Extended exposure to solvent extraction and the use of denaturing solvents such as ethanol and isopropanol impaired the protein solubility and emulsifying properties of the macauba kernel proteins. The MKM from *A. aculeata* showed a higher potential for producing protein ingredients, owing to its superior protein solubility, emulsifying activity, and emulsion stability compared to MKM from *A. totai*. Therefore, the oil extraction of macauba kernels was improved based on protein functional properties, yielding a deoiled meal suitable to produce functional protein-rich ingredients. Further research on the structure–function relationship of macauba kernel proteins could contribute to elucidating the differences in protein denaturation attained in specific oil extraction steps.

The recovery of macauba kernel proteins through sieve fractionation after oil extraction was also studied. Mild oil extraction treatments resulted in fractions enriched in soluble protein, as observed in preliminary sieving trials. The macauba kernel meal from oil extraction comprising hydraulic pressing, impact milling to 1 mm, and solvent extraction with hexane was selected for a representative sieving. This meal was sieve-fractionated with a cut size of 62 µm to produce a protein concentrate with a 65.6% protein content and a protein enrichment of 74.1% in relation to the selected macauba kernel meal. Compared to the MKM before sieving, this protein concentrate showed a reduced gelling concentration from 8 to 6% and an increased emulsion stability from 149 to 345 min. The determination of the protein composition and structure–function relationship might be addressed in future research to evaluate the differences in the functional properties between the protein concentrate and the MKM.

Therefore, the recovery of macauba kernel proteins through sieve fractionation after oil extraction resulted in a functional protein concentrate. The use of less intensive techniques, such as dry fractionation, proved to be a feasible option for valorizing the macauba kernel proteins. This could make macauba a novel source of protein-based ingredients, fostering the development of a value chain based on a circular bioeconomy.

## Figures and Tables

**Table 1:** Parameters used for obtaining the deoiled macauba kernel meals.

Meal Code	Processing Parameters			
	Pressing Method	Solvent	Macauba Species	Milling
SHA_1_	Screw Pressing	Hexane	*A. aculeata*	1 mm
HHA_1_	Hydraulic Pressing	Hexane	*A. aculeata*	1 mm
HHA_0.5_	Hydraulic Pressing	Hexane	*A. aculeata*	0.5 mm
HHA_2_	Hydraulic Pressing	Hexane	*A. aculeata*	2 mm
HEA_1_	Hydraulic Pressing	Ethanol	*A. aculeata*	1 mm
HIA_1_	Hydraulic Pressing	Isopropanol	*A. aculeata*	1 mm
HHT_1_	Hydraulic Pressing	Hexane	*A. totai*	1 mm

**Table 2:** Influence of oil extraction conditions on the oil content and functional properties of macauba kernel meals.

Meal Code	Processing Parameters (Pressing Method, Milling, Solvent, Macauba Species)	OC (% DM)	Functional Properties				
			PS (%)	WBC (mL/g DM)	OBC (mL/g DM)	EAI (m^2^/g protein)	ES (min)
SHA_1_	Screw pressing, 1 mm, hexane, *A. aculeata*	1.1 ± 0.2 ^b^	47.6 ± 1.2 ^c^	4.7 ± 0.1 ^b^	1.4 ± 0.0 ^e^	109.0 ± 2.0 ^c^	84.7 ± 13.5 ^c^
HHA_1_	Hydraulic pressing, 1 mm, hexane, *A. aculeata*	4.6 ± 0.3 ^a^	77.1 ± 0.7 ^a^	4.1 ± 0.1 ^cd^	2.2 ± 0.0 ^d^	181.0 ± 4.8 ^a^	149.3 ± 18.2 ^a^
HHA_0.5_	Hydraulic pressing, 0.5 mm, hexane, *A. aculeata*	4.2 ± 0.5 ^a^	78.1 ± 0.5 ^a^	3.8 ± 0.2 ^d^	2.2 ± 0.1 ^d^	179.7 ±3.0 ^a^	74.0 ± 8.9 ^cd^
HHA_2_	Hydraulic pressing, 2 mm, hexane, *A. aculeata*	4.6 ± 0.1 ^a^	74.9 ± 1.3 ^a^	4.1 ± 0.1 ^cd^	2.7 ± 0.1 ^b^	168.4 ± 2.7 ^a^	45.8 ± 0.7 ^de^
HEA_1_	Hydraulic pressing, 1 mm, ethanol, *A. aculeata*	4.7 ± 0.1 ^a^	64.4 ± 0.9 ^b^	4.3 ± 0.1 ^bc^	3.1 ± 0.0 ^a^	107.8 ± 1.3 ^c^	59.3 ± 4.1 ^cde^
HIA_1_	Hydraulic pressing, 1 mm, isopropanol, *A. aculeata*	5.3 ± 0.3 ^a^	79.3 ± 1.2 ^a^	4.3 ± 0.0 ^bc^	3.1 ± 0.0 ^a^	135.8 ± 7.8 ^b^	138.9 ± 0.9 ^b^
HHT_1_	Hydraulic pressing, 1 mm, hexane, *A. totai*	5.3 ± 0.4 ^a^	63.1 ± 0.7 ^b^	5.2 ± 0.1 ^a^	2.9 ± 0.0 ^b^	102.5 ± 2.0 ^c^	31.5 ± 0.7 ^e^

OC: oil content; DM: dry matter; PS: protein solubility; WBC: water-binding capacity; OBC: oil-binding capacity; EAI: emulsifying activity index; ES: emulsion stability. Values followed by different superscript letters in the same column are significantly different (*p* < 0.05) according to Tukey’s test.

**Table 3:** Composition of the fractions and dry fractionation performance parameters after the representative sieve fractionation of macauba kernel meal (*A. aculeata*) from hydraulic pressing, milling to 1 mm, and solvent extraction with hexane (HHA_1_).

Fraction	Oil Content (% DM)	Total Protein Content (% DM)	Yield (%)	Protein Separation Efficiency (%)	Protein Enrichment (%)
MKM	5.2 ± 0.2 ^b^	37.7 ± 0.2 ^b^	NA	NA	NA
500 µm	8.0 ± 0.0 ^a^	34.4 ± 0.1 ^d^	51.9 ± 1.4 ^a^	47.4	−8.7
250 µm	3.0 ± 0.3 ^c^	33.6 ± 0.0 ^e^	18.9 ± 0.9 ^b^	16.8	−10.9
150 µm	2.0 ± 0.0 ^d^	33.8 ± 0.2 ^e^	9.2 ± 0.5 ^c^	8.3	−10.2
100 µm	1.4 ± 0.1 ^de^	33.9 ± 0.1 ^e^	4.8 ± 0.2 ^d^	4.3	−10.1
62 µm	1.6 ± 0.0 ^d^	37.3 ± 0.1 ^c^	5.8 ± 0.3 ^d^	5.7	−1.0
FF (<62 µm)	0.7 ± 0.1 ^e^	65.6 ± 0.1 ^a^	9.4 ± 0.7 ^c^	16.4	74.1

DM: dry matter; MKM: macauba kernel meal; FF: fine fraction; NA: not applicable. Values followed by different superscript letters in the same column are significantly different (*p* < 0.05) according to Tukey’s test.

**Table 4:** Functional properties of the fractions after the representative sieve fractionation of macauba kernel meal.

Fraction	PS (%)	WBC (mL/g DM)	OBC (mL/g DM)	EAI (m^2^/g Protein)	ES (min)	LGC (%)
MKM	77.86 ± 2.46 ^b^	3.53 ± 0.11 ^c^	2.70 ± 0.01 ^d^	183.81 ± 8.26 ^a^	147.29 ± 4.79 ^c^	8.0 ± 0.0 ^b^
500 µm	87.62 ± 1.03 ^a^	3.43 ± 0.08 ^c^	2.52 ± 0.06 ^e^	180.22 ± 4.19 ^ab^	269.09 ± 6.40 ^b^	8.0 ± 0.0 ^b^
250 µm	71.22 ± 2.45 ^c^	3.84 ± 0.07 ^c^	2.97 ± 0.04 ^c^	175.71 ± 2.39 ^abc^	313.15 ± 2.83 ^ab^	10.0 ± 0.0 ^a^
150 µm	65.77 ± 0.70 ^d^	7.22 ± 0.23 ^a^	6.15 ± 0.03 ^a^	191.33 ± 3.18 ^a^	128.64 ± 0.48 ^cd^	8.0 ± 0.0 ^b^
100 µm	64.10 ± 0.31 ^d^	7.41 ± 0.19 ^a^	6.14 ± 0.04 ^a^	180.62 ± 3.18 ^a^	95.56 ± 0.1.05 ^d^	6.0 ± 0.0 ^c^
62 µm	64.61 ± 0.52 ^d^	5.11 ± 0.05 ^b^	4.45 ± 0.01 ^b^	164.12 ± 3.07 ^bc^	157.49 ± 1.22 ^c^	8.0 ± 0.0 ^b^
FF (<62 µm)	60.90 ± 0.60 ^e^	1.41 ± 0.03 ^d^	1.55 ± 0.07 ^f^	163.34 ± 1.80 ^c^	345.22 ± 6.88 ^a^	6.0 ± 0.0 ^c^

DM: dry matter; PS: protein solubility; WBC: water-binding capacity; OBC: oil-binding capacity; EAI: emulsifying activity index; ES: emulsion stability; LGC: least gelling concentration; MKM: macauba kernel meal; FF: fine fraction. Values followed by different letters in the same column are significantly different (*p* < 0.05) according to Tukey’s test.

## Data Availability

The data presented in this study are available on request from the corresponding author.
